# Evaluation of a Dual PI3K/mTOR Inhibitor PF-04691502 against Bladder Cancer Cells

**DOI:** 10.1155/2022/8110796

**Published:** 2022-06-24

**Authors:** Xiaosong Shang, Xinyu Na, Lei Wang, Zhiqin Yang, Pengpeng Ren

**Affiliations:** Department of Urology, Zhenhai People's Hospital, Ningbo, Zhejiang, China

## Abstract

Targeting the phosphatidylinositol-3 kinase/protein kinase B/mammalian target of rapamycin (PI3K/Akt/mTOR) signalling pathway is a promising strategy for the treatment of various cancers, including bladder cancer (BC). PF-04691502 is a relatively novel dual PI3K/mTOR inhibitor that exerts inhibitory effects against various cancer cells. However, the effects of PF-04691502 in BC cells have not been clarified thus far. This study aimed to evaluate the antitumour effects of PF-04691502 and the mechanisms underlying these antitumour effects in BC cells. The effects of PF-04691502 on the viabilities of BC cells were examined using the cell counting kit 8 (CCK-8) assay. Cell migration and invasion were measured using the wound healing assay and transwell assay, respectively. Cellular apoptosis was determined using flow cytometry. The change in the cellular protein levels was measured using western blotting. siRNA was used to study the role of PTEN in the antitumour effects of PF-04691502. PF-04691502 inhibited the proliferation, migration, and invasion of BC cells. Additionally, PF-04691502 induced apoptosis of BC cells via the intrinsic pathway. PF-04691502 inhibited the expression of Mcl-1 and the PI3K/Akt/mTOR pathway in BC cells. In addition, PF-04691502 increased the apoptosis induced by various chemotherapeutic agents in BC cells. Taken together, PF-04691502 could be used alone or in combination with other chemotherapeutic agents in the treatment of BC.

## 1. Introduction

Bladder cancer (BC) is a common urological system malignancy worldwide. More than 5,00,000 patients are estimated to be newly diagnosed with BC, and there are 2,00,000 BC-related deaths each year [1]. Approximately 75–80% of patients with newly diagnosed BC have non-muscle-invasive bladder cancer (NMIBC) and the remaining have BC that eventually develops into muscle-invasive disease [2]. Currently, the main treatment strategy for patients with high-risk NMIBC is intravesical instillation chemotherapy after transurethral resection of the tumour tissues [3]. However, two major obstacles influence the clinical outcome. Chemoresistance develops in approximately 70% of all BC patients. Further, interference of the physiological condition of the bladder often causes a poor utilisation rate of instillation agents [4]. Thus, it is necessary to unveil the mechanisms underlying BC and develop novel effective agents and therapeutic strategies.

Previous studies indicate that the phosphatidylinositol-3 kinase (PI3K)/Akt (protein kinase B)/mammalian target of rapamycin (mTOR) pathway plays an essential role in various biological activities such as cell migration, differentiation, angiogenesis, and cell death [5]. The PI3K/mTOR signalling pathway is an essential player in the tumorigenesis of BC and targeting the PI3K/Akt/mTOR pathway can be a potential therapeutic strategy for the treatment of BC. For example, atractylenolide I (ATR-1), a natural product, shows antitumour effects against BC via inhibition of the PI3K/Akt/mTOR pathway [6]. A previous study showed that a dual PI3K and mTOR inhibitor, NVP-BEZ325, significantly inhibits the tumorigenesis of BC cells both *in vitro* and *in vivo* [7]. Thus, targeting PI3K/Akt/mTOR might be a promising strategy for the treatment of BC.

Recently, a novel and promising PI3K/mTOR dual inhibitor PF-04691502 was developed, and it showed promising antitumour effects against various cancers such as colorectal cancer, B-cell non-Hodgkin's lymphomas, and head and neck cancer [8–10]. However, the effects of PF-04691502 against BC cells have not been investigated thus far. In this study, we examined the antitumour effects of PF-04691502 in BC cells and the mechanisms underlying these effects. Our results suggest that PF-04691502 showed strong inhibitory effects on BC cells and thus may be used as a potential agent for the treatment of BC.

## 2. Materials and Methods

### 2.1. Cell Culture and Chemicals

BC cell lines (T24 and 5637) were obtained from the Shanghai Bank of Cell Culture (Shanghai, China). Cells were cultured in RPMI1640 medium (Gibco, USA) supplemented with 10% foetal bovine serum (FBS, Gibco) and 100 U of penicillin/streptomycin (Gibco). Cells were maintained in a humidified incubator with 5% CO_2_ at 37°C. PF-04691502 was purchased from Selleck Chemicals Ltd. (USA). PF-04691502 was prepared as a 100 mM stock solution in dimethyl-sulphoxide (DMSO). The stock was stored at −20°C. All other chemicals were obtained from Sigma-Aldrich (USA) unless indicated specifically.

### 2.2. Cell Viability Assay

Cell viabilities were measured using the cell counting kit 8 (CCK-8) kit (Dojindo, Japan) according to the manufacturer's instructions. BC cells were seeded into 96-well plates at a density of 5 × 10^3^ cells/well. After different treatments, 10 *μ*L of CCK-8 was added into each well and the absorbance was read at 450 nm using a microplate reader (BioTek, USA).

### 2.3. Wound Healing Assay

BC cells were seeded into 6-well plates at a density of 1 × 10^5^ cells/well. When the cells reached around 90% confluence, the monolayer cells were scratched using a sterile pipette tip and were washed with phosphate-buffered saline (PBS) to remove the debris. Then, the cells were treated with various doses of PF-04691502 for 24 h, and the wound healing status was observed using an inverted microscope (Olympus, Japan).

### 2.4. Transwell Assay

Cellular invasion was determined using the transwell assay. Cells were collected and resuspended in a medium without FBS and added into the transwell chambers (Corning, USA) covered with Matrigel Matrix (Sigma-Aldrich, USA). The cells were treated with various doses of PF-04691502 for 24 h; then, 500 *μ*L of a complete medium was added into the lower chamber. After culture for another 24 h, the cells in the upper layer were wiped away using cotton swabs, and the cells in the lower layer were fixed with 4% ethanol and stained with 0.1% crystal violet (Solarbio, China). The invaded cells were observed under an inverted microscope (Olympus, Japan). We randomly selected 5 fields to calculate the average number of invaded cells.

### 2.5. Cellular Apoptosis Assay

BC cells were seeded into 6-well plates at a density of 1 × 10^5^ cells/well. After different treatments, the cells were collected and stained with the annexin V and propidium iodide (PI) staining kit (Sigma, USA) according to the manufacturer's guide. Cellular apoptosis was measured using a FACSCanto II flow cytometer (BD Biosciences, USA).

### 2.6. Cellular Transfection

The empty vector (pcDNA3.1, EV) and pcDNA3.1-Mcl-1 (OV Mcl-1) were obtained from GenePharma (China). The myr-Akt vector was purchased from Addgene (USA). siRNA against PTEN (si-PTEN) and negative scramble control (si-NC) were purchased from RioBio Ltd. (China). The transfection was conducted using lipofectamine 2000 (Life Technologies, USA) according to the manufacturer's instructions. The transfection efficiency was evaluated using western blotting.

### 2.7. Caspase-3 Activities Assay

The activities of caspase-3 were measured using the caspase-3 activity kit (Abcam, USA) according to the manufacturer's instructions. The changes in optical density (OD) were measured at an excitation wavelength of 400 nm. The results are expressed as fold changes relative to the control.

### 2.8. Western Blots

After different treatments, BC cells were collected and washed with PBS. Then, the cells were lysed with RIPA buffer (Cellular Signalling Technologies, USA). The protein concentrations were measured using the BCA kit (Beyotime, China) according to the manufacturer's instructions. Then, 20 *μ*g of protein was subjected to 10% sodium dodecyl sulphate polyacrylamide gel electrophoresis (SDS-PAGE) and transferred to a polyvinylidene fluoride (PVDF) membrane (Millipore, USA). The membrane was blocked with 5% skimmed milk for 1 h at room temperature, and the membrane was incubated overnight at 4°C with a primary antibody. After washing three times with TBST, the membrane was incubated with the corresponding secondary antibody at room temperature for 1 h. The results of western blotting were visualised using the enhanced chemiluminescence (ECL) technique.

### 2.9. Statistical Analyses

Statistical analyses were performed using SPSS 14.0 software (SPSS Inc., Chicago, IL, USA). Data are expressed as the mean ± standard deviation (SD). Differences among groups were determined using a one-way analysis of variance (ANOVA) followed by a Tukey's honestly significant difference (HSD) test. A *P* value less than 0.05 was considered significantly different.

## 3. Results

### 3.1. PF-04691502 Inhibits the Proliferation, Migration, and Invasion of BC Cells

**Figure 1:**
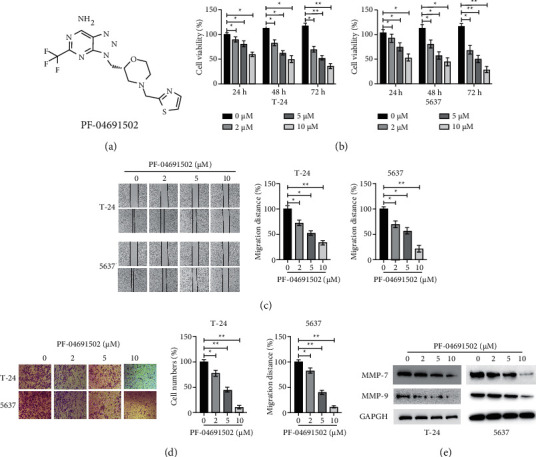
PF-04691502 inhibits the viability, migration, and invasion of bladder cancer cells. (a) The chemical structure of PF-04691502. (b) T-24 and 5637 cells were treated with various doses of PF-04691502 for the specific times, and then, cellular viabilities were measured. (c) T-24 and 5637 cells were treated with various doses of PF-04691502 for 24 h and cellular migration distance was measured. (d) T-24 and 5637 cells were treated with various doses of PF-04691502 for 24 h and cellular invasion was measured. (e) T-24 and 5637 cells were treated with various doses of PF-04691502 for 24 h and the levels of the specific proteins were measured using western blotting. Data are representative of at least three independent experiments (^*∗*^*P* < 0.05; ^*∗∗*^*P* < 0.01).

We evaluated the antitumour effects of PF-04691502 (Figure 1(a)) in two BC cell lines T24 and 5637. CCK-8 assays showed that PF-04691502 affected the viability of both BC cell lines in a dose- and time-dependent manner (Figure 1(b)). Additionally, wound healing assay and transwell assay indicated that PF-04691502 inhibited the migration and invasion of BC cells in a dose-dependent manner (Figures 1(c) and 1(d)). Further, treatment with PF-04691502 inhibited the expression of matrix metalloproteinase-7 (MMP-7) and MMP-9 in BC cells in a dose-dependent manner (Figure 1(e)). Taken together, these results suggest that PF-04691502 exerts antitumour effects against BC cells.

### 3.2. PF-04691502 Induces Apoptosis via the Intrinsic Pathway in BC Cells

**Figure 2:**
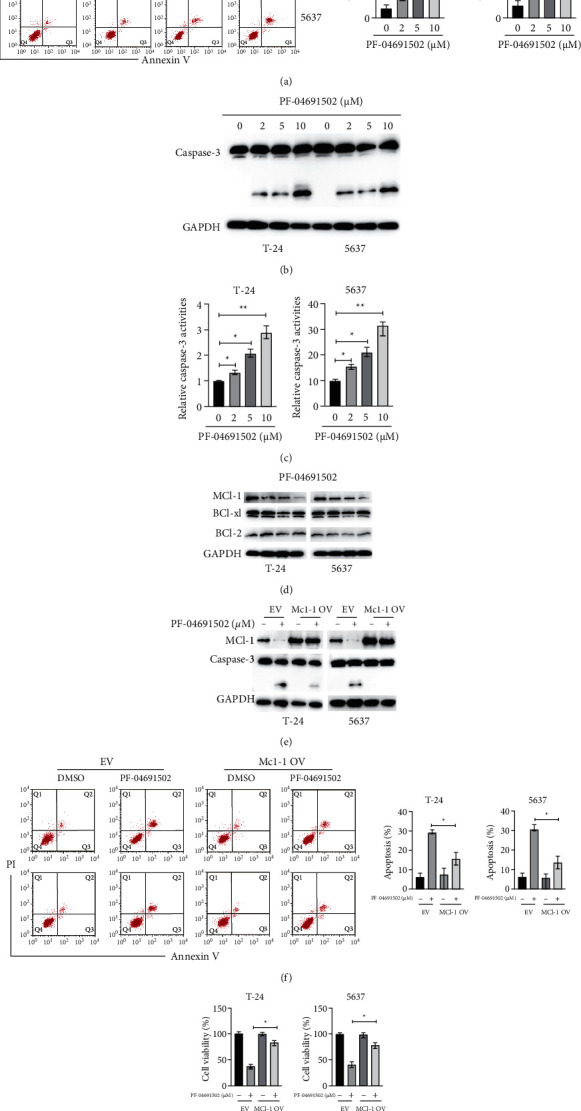
PF-04691502 induces apoptosis via downregulation of Mcl-1 in bladder cancer cells (a) T-24 and 5637 cells were treated with the indicated doses of PF-04691502 for 24 h and then, cellular apoptosis was measured using flow cytometry. (b) T-24 and 5637 cells were treated with the indicated doses of PF-04691502 for 24 h and cellular lysates were subjected to western blot analysis. (c) T-24 and 5637 cells were treated with the indicated doses of PF-04691502 for 24 h and caspase-3 activities were measured. (d) T-24 and 5637 cells were treated with the indicated doses of PF-04691502 for 24 h and the levels of the specific proteins were measured using western blotting. (e) T-24 and 5637 cells were transfected as indicated for 12 h and then, the cells were treated with or without PF-04691502 for another 24 h and the levels of the specific proteins were measured using western blotting. (f) T-24 and 5637 cells were treated as described above, and cellular apoptosis was measured. (g) T-24 and 5637 cells were treated as described above, and cellular viabilities were measured. Data are representative of at least three independent experiments (^*∗*^*P* < 0.05; ^*∗∗*^*P* < 0.01).

To further evaluate the antitumour effects of PF-04691502 in BC cells, we measured cellular apoptosis. BC cells were treated with various doses of PF-04691502 for 24 h, and PF-04691502-induced apoptosis in BC cells in a dose-dependent manner (Figure 2(a)). Results of western blotting showed that treatment with PF-04691502-induced cleavage of caspase-3 in BC cells in a dose-dependent manner (Figure 2(b)). Furthermore, PF-04691502 treatment increased the activities of caspase-3 in a dose-dependent manner in BC cells (Figure 2(c)). Results of a previous study showed that proteins in the Bcl-2 family play essential roles in apoptosis [11]. Thus, we examined the effects of PF-04691502 on the levels of Bcl-2 family proteins in BC cells. Our results showed that PF-04691502 did not have a marked effect on the expression of Bcl-2, Bcl-xl, and Bax in BC cells (Figure 2(d)). Interestingly, PF-04691502 treatment downregulated the expression of Mcl-1 in a dose-dependent manner in BC cells (Figure 2(d)). To clarify the role of Mcl-1 in PF-04691502-mediated apoptosis, we transfected BC cells with a vector overexpressing Mcl-1 (Figure 2(e)). Further, forced expression of Mcl-1 significantly reduced the cleavage of caspase-3 caused by PF-04691502 in BC cells (Figure 2(e)). Moreover, upregulation of Mcl-1 significantly reduced the apoptosis induced by PF-04691502 in BC cells (Figure 2(f)). In addition, the viability of BC cells markedly increased after treatment with PF-04691502 after forced expression of Mcl-1 (Figure 2(g)). Taken together, these results suggest that PF-04691502 induces apoptosis by decreasing Mcl-1 levels and through the intrinsic pathway in BC cells.

### 3.3. PF-04691502 Inhibits the PI3K/Akt/mTOR Pathway in BC Cells

**Figure 3:**
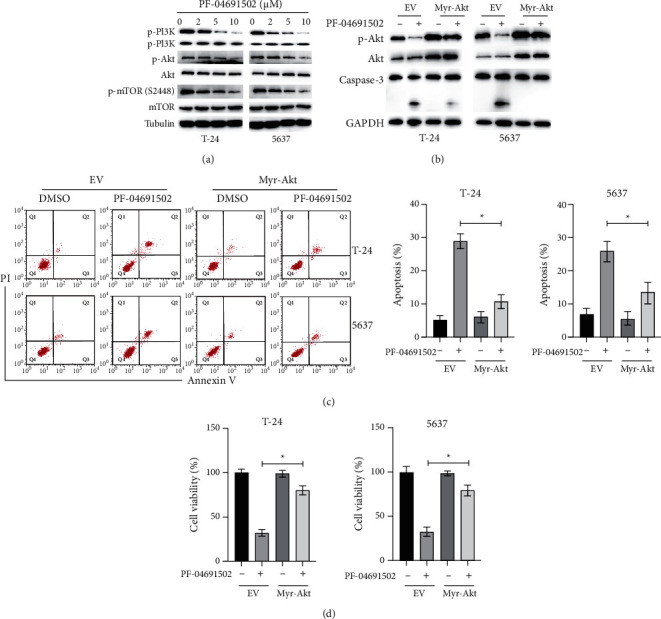
PF-04691502 inhibits the PI3K/Akt/mTOR pathway in bladder cancer cells. (a) T-24 and 5637 cells were treated with the indicated doses of PF-04691502 for 24 (h) and the levels of specific proteins were measured using western blot. (b) T-24 and 5637 cells were transfected with an empty vector (EV) or a vector expressing myr-Akt (OV Akt) for 12 h; then, the cells were treated with or without PF-04691502 for another 24 h and the specific proteins were measured using western blots. (c) T-24 and 5637 cells were treated as indicated above, and cellular apoptosis was measured. (d) Cellular viabilities of the cells were measured. Data are representative of at least three independent experiments (^*∗*^*P* < 0.05; ^*∗∗*^*P* < 0.01).

We examined the effects of PF-04691502 on the PI3K/Akt/mTOR pathway in BC cells. Our results showed that treatment with PF-04691502 successfully inhibited the PI3K/Akt/mTOR pathway in BC cells (Figure 3(a)). To evaluate the role of the PI3K/Akt/mTOR pathway in the antitumour effects of PF-04691502, BC cells were transfected with a vector constitutively expressing activated myristoylated Akt (myr-Akt) according to a previous study [6]. Forced expression of myr-Akt inhibited the PF-04691502-induced cleavage of caspase-3 in BC cells (Figure 3(b)). Additionally, flow cytometry analysis confirmed that overexpression of myr-Akt reduced the apoptosis of BC cells caused by PF-04691502 (Figure 3(c)). Moreover, CCK-8 assays showed that overexpression of myr-Akt significantly increased the viability of BC cells after treatment with PF-04691502 (Figure 3(d)). Taken together, these data indicate that PF-04691502 exerts its antitumour effects against BC cells via inhibition of the PI3K/Akt/mTOR pathway.

### 3.4. PF-04691502 Induces Upregulation of PTEN, Which Is Essential for the Inhibition of the Akt Signalling Pathway

**Figure 4:**
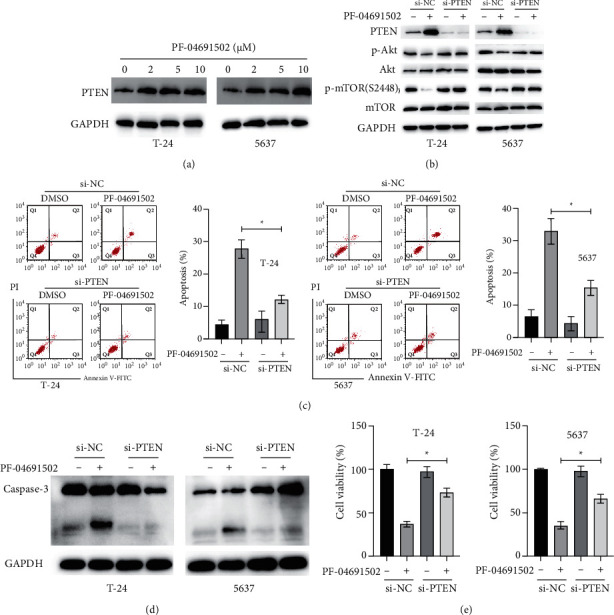
PF-04691502 induces upregulation of PTEN, which is responsible for the inhibition of the PI3K/Akt/mTOR pathway in bladder cancer cells. (a) T-24 and 5637 cells were treated with the indicated doses of PF-04691502 for 24 h and the levels of PTEN were measured using western blotting. (b) T-24 and 5637 cells were transfected with scrambled negative control (si-NC) or si-PTEN for 12 h and then, the cells were treated with or without PF-04691502 for another 24 h and cellular lysates were subjected to western blot analysis with indicated antibodies. (c) T-24 and 5637 cells were treated as described above, and cellular apoptosis was measured. (d) T-24 and 5637 cells were treated as described above, and caspase-3 levels were measured. (e) T-24 and 5637 cells were treated as described above, and cellular viabilities were measured. Data are representative of at least three independent experiments (^*∗*^*P* < 0.05; ^*∗∗*^*P* < 0.01).

Several studies showed that the lack of PTEN, which is a regulator of the PI3K/Akt pathway, is a common genetic cause of BC [12]. Therefore, we examined the status of PTEN in BC cells after treatment with PF-04691502. Treatment with PF-04691502 upregulated PTEN in a dose-dependent manner in BC cells (Figure 4(a)). To investigate the role of PTEN in the antitumour effects of PF-04691502, siRNA against PTEN was applied to knock down *PTEN* in BC cells (Figure 4(b)). Silencing of PTEN abrogated the inhibitory effects of PF-04691502 on the PI3K/Akt/mTOR pathway in BC cells (Figure 4(b)). Knockdown of *PTEN* attenuated the apoptosis and cleavage of caspase-3 caused by PF-04691502 in BC cells (Figures 4(a) and 4(d)). Additionally, knockdown of *PTEN* increased the viabilities of BC cells after treatment with PF-04691502 (Figure 4(e)). Taken together, our findings suggest that PF-04691502–induced upregulation of PTEN is critical for the inhibition of the PI3K/Akt/mTOR pathway in BC cells.

### 3.5. PF-04691502 Synergistically Induces Apoptosis with Various Chemotherapeutic Agents in BC Cells

**Figure 5:**
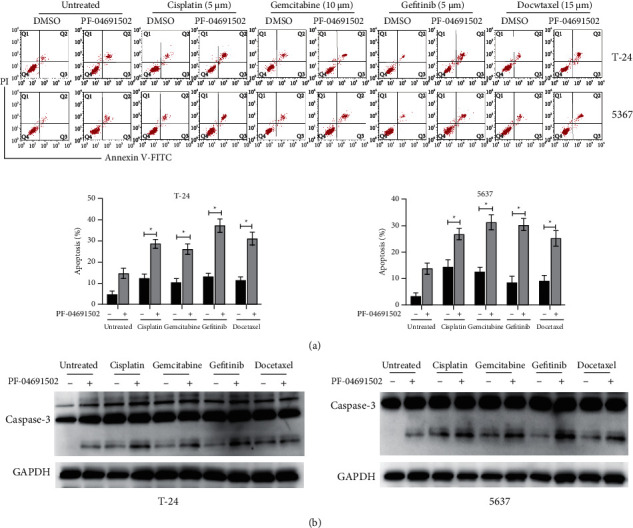
PF-04691502 enhances the apoptosis induced by various chemotherapeutic agents in bladder cancer cells. (a) T-24 and 5637 cells were treated as indicated for 24 h and cellular apoptosis was measured. (b) T-24 and 5637 cells were treated as indicated for 24 h and levels of caspase-3 were measured using western blotting. Data are representative of at least three independent experiments (^*∗*^*P* < 0.05).

We examined the effects of PF-04691502 in combination with various chemotherapeutic agents in BC cells. Low doses of PF-04691502 (2 *μ*M) significantly increased the apoptosis induced by various chemotherapeutic agents (cisplatin, 5 *μ*M; gemcitabine, 10 *μ*M; gefitinib, 5 *μ*M; docetaxel 15 *μ*M) in BC cells (Figure 5(a)). Moreover, the results of western blotting showed that PF-04691502 administered in combination with various chemotherapeutic agents induced more cleavage of caspase-3 than individual agents in BC cells (Figure 5(b)). These results indicate that PF-04691502 has a synergistic effect with different chemotherapeutic agents against BC cells.

## 4. Discussion

Although great progress has been made in the treatment of BC, the overall prognosis of patients continues to be poor. Therefore, it is necessary to develop novel agents and therapeutic strategies. Previous studies indicate that dysregulation of the PI3K/Akt/mTOR pathway plays an essential role in the tumorigenesis of BC and targeting the PI3K/Akt/mTOR pathway is a potential therapeutic strategy for BC. To date, the effects of several PI3K/Akt/mTOR inhibitors have been examined clinically in BC, such as everolimus, GSK2126458, and buparlisib [13–15]. PF-04691502 is a novel dual PI3K/mTOR inhibitor that has antitumour effects against various cancers. However, the effect of PF-04691502 in BC cells has not been clarified thus far. In this study, we evaluated the effects of PF-04691502 in BC cells and the mechanism underlying these effects.

Our results showed that PF-04691502 inhibited the proliferation, migration, and invasion of BC cells. These findings are in line with those reported in previous studies in that PF-04691502 had antitumour effects against various cancer cells such as head and neck cancer cells, non–small-cell lung cancer cells, and colorectal cancer cells [9, 16, 17]. MMP-7 and MMP-9 belong to the MMP family and are capable of cleaving extracellular matrix proteins (ECMs), which participate in various tumorigenesis activities such as tumour progression and metastasis [18]. MMP-7 is the smallest member of the MMP family. Plasma MMP-7 levels are much higher in BC patients at a high risk of disease progression [19]. Serum levels of MMP-7 can be applied as an independent prognostic factor in patients with locally advanced and/or metastatic BC [20]. In addition, high expression of MMP-9 correlates with an unfavourable prognosis of BC [21]. We found that treatment with PF-04691502 downregulated MMP-7 and MMP-9. Our findings are in line with those reported previously in that many PI3K/mTOR inhibitors decrease the expression of MMPs [22]. Considering the vital role of MMPs in the metastasis of tumours, it would be intriguing to investigate the inhibitory effects of PF-04691502 on the metastasis of BC both *in vitro* and *in vivo*.

Apoptosis is a well-orchestrated process, and it is an important regulator of cell differentiation, development, and cell death. The two pathways leading to apoptosis are the extrinsic and intrinsic pathways [6]. Most chemotherapeutic agents induce apoptosis via the intrinsic pathway, which is strictly subjected to regulation by the Bcl-2 family of proteins [23]. In this study, we found that treatment with PF-04691502 downregulated Mcl-1. In addition, forced expression of Mcl-1 significantly abrogated the antitumour effects of PF-04691502 against BC cells. These findings suggest that PF-04691502 induces apoptosis via the intrinsic pathway and is dependent on the downregulation of Mcl-1. Although Mcl-1 was described as an oncogene in many cancers, to date, limited knowledge is available about the role of Mcl-1 in the tumorigenesis of BC. Many dual PI3K/mTOR inhibitors exerted their antitumour effects via the inhibition of Mcl-1. For instance, the dual PI3K/mTOR inhibitor PKI-402 inhibits the growth of ovarian cancer cells via degradation of Mcl-1 [24]. Another study showed that the dual PI3K/mTOR inhibitor NVP-BEZ235 downregulated Mcl-1 and thereby inhibited the growth of ovarian cancer cells [25]. To the best of our knowledge, our findings showed for the first time that inhibition of Mcl-1 is critical for the antitumour effects of PF-04691502, and further studies should be performed to confirm this finding in more cancer cell types.

Our results showed that inhibition of the PI3K/Akt pathway is the mechanism underlying the downregulation of Mcl-1. This result is consistent with that reported previously. For instance, the PI3K/Akt pathway promotes the stability of Mcl-1 via inhibition of the phosphorylation of Mcl-1 [26]. Additionally, we observed that PF-04691502 treatment upregulated PTEN, which is essential for the inhibition of PI3K/Akt in BC cells. Some studies suggest that PF-04691502 inhibits the growth of tumour cells regardless of the status of PTEN. For example, PF-04691502-induced apoptosis in PTEN-null ovarian cancer cells [27]. PF-04691502 continued to prolong the survival of *PTEN*-deficient mice in an animal model of head and neck cancer although to a lesser extent than that of the wild-type mice [16]. These discrepancies may be because of different cancer cell types. Therefore, the relationship between PTEN and the antitumour effects of PF-04691502 should be further investigated.

The high mortality associated with BC is largely related to its recurrence because of the development of chemoresistance. Many studies reported that chemoresistance is closely correlated with the activation of the PI3K/Akt/mTOR pathway in cancer cells [28]. To date, several studies reported that various PI3K/Akt/mTOR inhibitors could overcome chemoresistance in many cancer cells such as BEZ235, CMG002, and PKI-402 [24, 29, 30]. Additionally, PF-04691502 overcame chemoresistance in gefitinib- and erlotinib-resistant non–small-cell lung cancer cells [27]. Our results were consistent with those reported previously in that we showed that PF-04691502 enhanced the apoptosis induced by various chemotherapeutic agents in BC cells. Thus, PF-04691502 can be used alone or in combination with other agents in the treatment of cancers.

Our study has some limitations. We only evaluated the antitumour effects of PF-04691502 *in vitro*, and the effects of PF-04691502 in xenograft mice models should be evaluated in future studies. Further, although we showed that PF-04691502 downregulated Mcl-1, it could be caused by degradation or inhibition of translation. Thus, it would be interesting to further examine the mechanism underlying Mcl-1 downregulation. We only focused on the effects of PF-04691502 on the PI3K/Akt/mTOR pathway, and further studies should be performed to determine the other pathways that may be involved in the effects of PF-04691502.

In conclusion, our results showed that PF-04691502 inhibits tumorigenesis and induces apoptosis via the intrinsic pathway in BC cells. Examination of the mechanism underlying the effects of PF-04691502 showed that PF-04691502 exerts its antitumour effects via the upregulation of PTEN, which negatively regulates the PI3K/Akt/mTOR pathway in BC cells. Considering the vital role of the PI3K/Akt/mTOR pathway in the tumorigenesis of BC, PF-04691502 might be used as a potential agent for the treatment of BC.

## Data Availability

All data generated or analysed during this study are included in this article. Further enquiries can be directed to the corresponding author.
